# A robust VMAT delivery solution for single‐fraction lung SABR utilizing FFF beams minimizing dosimetric compromise

**DOI:** 10.1002/acm2.12919

**Published:** 2020-05-29

**Authors:** Alex Burton, Keith Offer, Nicholas Hardcastle

**Affiliations:** ^1^ Peter MacCallum Cancer Centre Melbourne VIC Australia; ^2^ Centre for Medical Radiation Physics University of Wollongong Wollongong NSW Australia

**Keywords:** FFF, interplay, lung, robust, SABR, VMAT

## Abstract

Peripheral lung lesions treated with a single fraction of stereotactic ablative body radiotherapy (SABR) utilizing volumetric modulated arc therapy (VMAT) delivery and flattening filter‐free (FFF) beams represent a potentially high‐risk scenario for clinically significant dose blurring effects due to interplay between the respiratory motion of the lesion and dynamic multi‐leaf collimators (MLCs). The aim of this study was to determine an efficient means of developing low‐modulation VMAT plans in the Eclipse treatment planning system (v15.5, Varian Medical Systems, Palo Alto, USA) in order to minimize this risk, while maintaining dosimetric quality. The study involved 19 patients where an internal target volume (ITV) was contoured to encompass the entire range of tumor motion, and a planning target volume (PTV) created using a 5‐mm isotropic expansion of this contour. Each patient had seven plan variations created, with each rescaled to achieve the clinical planning goal for PTV coverage. All plan variations used the same field arrangement, and consisted of one dynamic conformal arc therapy (DCAT) plan, and six VMAT plans with varying degrees of modulation restriction, achieved through utilizing different combinations of the aperture shape controller (ASC) in the calculation parameters, and monitor unit (MU) objective during optimization. The dosimetric quality was assessed based on RTOG conformity indices (CI100/CI50), as well as adherence to dose–volume metrics used clinically at our institution. Plan complexity was assessed based on the modulation factor (MU/cGy) and the field edge metric. While VMAT plans with the least modulation restriction achieved the best dosimetry, it was found that there was no clinically significant trade‐off in terms of dose to organs at risk and conformity by reducing complexity. Furthermore, it was found that utilizing the ASC and MU objective could reduce plan complexity to near‐DCAT levels with improved dosimetry, which may be sufficiently robust to overcome the interplay effect.

**Abbreviations**
AIP: average intensity projection
API: application programming interface
ASC: aperture shape controller
DCAT: dynamic conformal arc therapy
EM: edge metric
FFF: flattening filter‐free
hVMAT: hybrid volumetric modulated arc therapy
ITV: internal target volume
MF: modulation factor
MIP: maximum intensity projection
MLC: multileaf collimator
MU: monitor unit
OAR: organ at risk
PTV: planning target volume
RTOG: Radiation Therapy Oncology Group
SABR: stereotactic ablative body radiotherapy
TB: TrueBeam
TBSTx: TrueBeam STx
VMAT: volumetric modulated arc therapy

## 1 Introduction

Volumetric modulated arc therapy (VMAT) provides modulation via continuous multileaf collimator (MLC) motion and dose rate modulation during gantry rotation.^1^, ^2^ This complex delivery is derived through inverse planning, which provides the ability to tune the treatment plan dosimetry. Although modulated delivery is best utilized for complex target‐organ at risk geometry, VMAT is used for a range of treatment sites throughout the body.^3^, ^4^, ^5^ As an example, peripheral lung stereotactic ablative body radiotherapy (SABR) volumes are typically not complex in shape and in many cases are not proximal to serial organs at risk, so there is little indication to create modulated plans. The main benefits of using VMAT in this context are highly efficient delivery and in general a more efficient planning process compared with 3D conformal treatments which require manual tuning of beam weights. The main benefits of VMAT in lung SABR thus may be the use of variable dose rate and gantry speeds that are produced by the optimizer, rather than complex aperture shapes.

When delivering modulated treatments to moving targets, there is a potential risk of interplay between tumor and MLC motion. Extensive research has shown this has limited impact in conventional fractionation due to the effects “blurring out” over the course of treatment; however, when using SABR which is typically delivered in 1–5 fractions this fractionation benefit may not hold.^6^, ^7^ Interplay‐induced dose discrepancies increase with decreasing number of breathing cycles completed by the patient during the treatment beam‐on time.^6^, ^7^, ^8^ Reducing aperture complexity,^7^, ^8^ increasing the number of beams and fractions,^6^, ^8^ reducing the dose rate,^8^, ^9^ and treating with higher dose per fraction have been shown to reduce the risk of clinically significant interplay effects.^6^, ^8^, ^10^ Furthermore, some studies also suggest that the risk of clinically significant interplay effects increase with tumor motion amplitude.^9^, ^11^ Limited data exists, however, for flattening filter‐free (FFF) dose rates and single fractions >18 Gy.^6^, ^7^, ^8^, ^10^ This means that single fraction lung cases treated with FFF beams represent a potentially high‐risk scenario, and that reductions in aperture complexity are highly desirable to retain the benefits of VMAT while limiting the dosimetric impact. The aim of this planning study was to determine an efficient means of developing low‐modulation VMAT plans in order to minimize the risk of interplay for single fraction lung SABR with high dose‐rate FFF beams.

## 2 Materials and methods

We selected 19 sequential lung SABR patients treated with a single 28 Gy fraction at our institution. Each patient was simulated using a 4DCT, with an internal target volume (ITV) contoured on the average intensity projection (AIP) while referencing the individual phases of the 4DCT and the maximum intensity projection (MIP). The AIP was used for contouring, planning, and dose computation. ITV volumes ranged from 0.3 to 20.3 cm^3^ (average 4.8 cm^3^), and the planning target volume (PTV) was created by adding an isotropic 5 mm margin to the ITV. All treatment planning was performed in the Eclipse treatment planning system (v15.5, Varian Medical Systems, Palo Alto, USA) using the photon optimizer (v15.5). Dose calculation was performed using the AcurosXB algorithm reporting dose to medium (v15.5).

Treatment plans using three arcs were created for each patient. The selected beam arrangement ensured that both dosimetrically acceptable dynamic conformal arc therapy (DCAT) and VMAT plans were produced. Two coplanar 210° arcs on the ipsilateral side (typical of VMAT), plus a 60° anterior noncoplanar arc delivered at couch 90° (typical of DCAT at our institution) were used, with the isocenter placed at the center of the target. Each plan used unique collimator angles for each beam, ranging from ±5°to 45°. In each patient, all plan types used identical jaw settings. All plans were rescaled to match the coverage requirement of 99% of the PTV covered by prescription dose (PTV D99% = 100%). All plans were developed using the clinical 6 MV FFF beam model allowing up to 1400 MU/min, for a Varian TrueBeam (TB) or TrueBeam STx (TBSTx), with the Millennium 120 MLC (5 mm central leaves) or Millennium HD 120 MLC (2.5 mm central leaves), respectively.

The optimization parameters tested to reduce modulation complexity were the aperture shape controller (ASC) and the monitor unit (MU) objective. The ASC defines aperture complexity by constraining the difference in allowable positions for adjacent leaves. There are six settings ranging from “None” (no constraints) to “Very High” (maximum constraint), which must be applied prior to entering the optimizer. The MU objective can be applied in the optimization environment and allows the user to define a minimum and maximum target MU for the plan. A penalty is applied to the optimizer cost function if the planned MU are not within the defined range, and this penalty is weighted by the “strength” assigned to the MU objective (a value between 0 and 100). This allows the MU objective to be used as a tool for creating efficient plan delivery, and therefore, its success was measured based on the level of reduction to plan complexity, rather than ability to achieve the a specific target MU. The application of these parameters was compared to a previously established method of modulation reduction known as “hybrid VMAT” (hVMAT).

Each plan variation was created from an initial VMAT plan developed following standard institutional procedure in order to establish the appropriate optimization objectives, to be used in all subsequent VMAT plans. Jaws were set by first fitting the MLC to the PTV with a 3 mm margin, and then using the Varian recommended jaw settings which positions them 2 mm from the most extended leaf. These plans used the institutional default ASC setting of “Moderate.” Other ASC settings were applied by copying this plan, changing the setting, and reoptimizing from new with all other objectives and settings held constant, and no user interaction. The DCAT plan was created by copying the first VMAT plan, replacing the MLC with conformal MLCs, and recalculating the dose using the VMAT field weights. In order to apply the MU objective, a two stage optimization was performed. That is, a VMAT plan (with the desired ASC setting) was used as the starting point, before reoptimizing with the desired MU objective applied, selecting the “continue optimization using current plan as base dose” option (which only proceeds through multiresolution levels 3 and 4 of optimization) with no further user interaction.. The hVMAT plan was created using the DCAT plan as the starting point, before reoptimizing, and once again selecting the “continue optimization” option with no further user interaction. This has a similar effect to applying the MU objective because the DCAT plan used as a starting point has significantly less MU than a standard VMAT. The hVMAT method for modulation suppression, however, requires both an initial VMAT plan (to provide the optimization objectives), and a DCAT plan (to provide the target MU), and is therefore a less efficient process than utilizing the MU objective.

Analysis was performed on a total of 19 sets of plans — 9 using the TBSTx model and 10 using the TB model. The optimum MU objective settings (maximum MU = 40% of original, strength = 80) were determined by trialing a broad combination of settings on the TBSTx patients (see Table A1, Appendix A). Note that the input maximum MU is determined as a fraction (%) of the original MU. These were the most penalizing settings trialed, and were selected to assess the maximum plan quality detriment as a result of modulation reduction. The MU objective was applied in combination with two ASC settings (“Moderate” and “Very High”). The DCAT and ASC = “None” VMAT plans were included for comparison to the least and most modulated plans, respectively, and the ASC = “None” plan was used as the benchmark case for dosimetric comparison. All plan types were renormalized to achieve the target coverage goal of PTV D99% = 100%.

Target doses were evaluated using the ITV D2% (as normalization was performed using coverage). The dose conformity was assessed using the RTOG 100% and 50% conformity indices (CI100 and CI50, respectively). The lung V5Gy, V20Gy, chest wall D30cc, chest wall D0.5cc, spinal canal D0.5cc, esophagus, D0.5cc and skin D0.5cc were also measured, and compared to the dose‐volume limits used in a recent clinical trial.^12^ Plan complexity/robustness was evaluated using the modulation factor (MF) defined as the monitor units per cGy (MU/cGy) and the edge metric (EM). The EM utilized was based on the aperture complexity metric described in reference,^13^ applying the recommended weight of zero to the leaf ends when calculating the aperture perimeter. A lower EM means reduced complexity, but is not strictly a measure of aperture openness. All dosimetric and plan complexity metrics were extracted using the Eclipse Scripting Application Programming Interface (API).

The average and standard deviation of each endpoint were determined for each plan type. Furthermore, the average difference of each metric from the benchmark plan (VMAT_noASC) was determined for each plan type, and a paired Student's *t*‐test was performed to determine the statistical significance of this difference. Additionally, the robustness metrics (EM and MF) for each plan type were compared to the DCAT plan using a paired Student's *t*‐test. The analysis was performed grouping both the TB and TBSTx data together; however, an assessment of the difference between the two models was also performed.

## 3 Results

Table 1 presents the mean (±1SD) outcome for each metric alongside the clinical planning goal, for each plan type. Each plan type was compared with the benchmark plan (VMAT_noASC) via a paired Student's *t*‐test (*P* < 0.05). The ITV D2% was lower than the benchmark case for the DCAT, MU objective, and hVMAT plans, with the maximum difference occurring in the DCAT plans. Similarly, the same four test plans had larger conformity indices, while the VMAT plans utilizing different ASC settings alone did not. On average, OAR dose–volume metrics in the test plans were higher than for the benchmark plan, but none of these differences were statistically significant. For every plan type, the EM and MF were significantly lower than the benchmark case (with the exception of the MF for the VMAT with “Moderate” ASC).

**Table 1:** Average (±1SD) for each tested outcome across all plan types.

Metric	Planning goal	VMAT noASC Avg (±1SD)	VMAT_mod Avg (±1SD)	VMAT_Vhigh Avg (±1SD)	DCAT Avg (±1SD)	Mod_40_80 Avg (±1SD)	Vhigh_40_80 Avg (±1SD)	hVMAT Avg (±1SD)
ITV D2% (Gy)	>35 <40	35.74 ±1.27	35.73 ±1.15	35.79 ±1.15	34.63 ± 1.36*	34.94 ±1.03*	35.42 ±1.32	35.04 ± 1.03*
RTOG CI100	<1.2‐1.3	1.12 ±0.06	1.12 ±0.06	1.13 ±0.06	1.67 ±0.28*	1.14 ±0.13	1.28 ±0.17*	1.26 ±0.13*
RTOG CI50	ALARA (<~6)	4.97 ±0.78	5.05 ±0.75	5.19 ±0.78	7.61 ±1.45*	5.71 ±1.4*	6.52 ±1.36*	6.50 ±1.4*
Lungs V5Gy (%)	<60	7.30 ±4.1	7.41 ±4.13	7.52 ±4.15	8.66 ±4.55	7.90 ±4.42	8.32 ±4.14	8.27 ±4.42
Lungs V20Gy (%)	<20	0.92 ±0.61	0.94 ±0.63	0.96 ±0.64	1.38 ±0.88	1.05 ±0.76	1.16 ±0.66	1.18 ±0.76
ChestWall D30cc (Gy)	<30	7.94 ±3.54	8.00 ±3.63	7.98 ±3.61	9.80 ±4.67	8.31 ±3.96	8.69 ±3.69	8.83 ±3.96
ChestWall D0.5cc (Gy)	<28	22.38 ±9.13	22.30 ±9.23	22.40 ±9.21	25.47 ± 10.05	21.97 ±8.78	23.04 ±9.05	22.91 ± 8.78
SpinalCanal D0.5cc (Gy)	<12	2.59 ±1.08	2.74 ±1.23	2.84 ±1.22	2.92 ±1.13	2.80 ±1.07	2.73 ±1.12	2.73 ±1.07
Esophagus D0.5cc (Gy)^a^	<15.4	2.70 ±1.51	2.80 ±1.39	2.83 ±1.49	2.82 ±1.43	2.56 ±1.27	2.62 ±1.41	2.62 ±1.27
Skin D0.5cc (Gy)	<24	8.46 ±2.69	8.26 ±2.75	8.38 ±2.6	10.10 ±3.5	8.46 ±3.23	8.87 ±2.72	9.36 ±3.23
MF (MU/cGy)	NA	2.97 ±0.39*^,Δ^	2.85 ±0.39*^,Δ^	2.69 ±0.37*^,Δ^	1.68 ±0.15*	2.11 ±0.22*^,Δ^	1.96 ±0.20*	1.87 ±0.20*
EM	NA	0.21 ±0.04^Δ^	0.18 ±0.03*^,Δ^	0.15 ±0.03*^,Δ^	0.06 ±0.01*	0.10 ±0.02*^,Δ^	0.07 ±0.02*	0.08 ±0.02*

Note

that this data is averaged across both beam models. A statistically significant difference to the VMAT_noASC result is indicated by an asterisk (*). For the robustness metrics, a statistically significant difference to the DCAT result is shown by a delta (^Δ^).

^a^ Only 13 cases had the esophagus contoured.

**Fig. 1:**
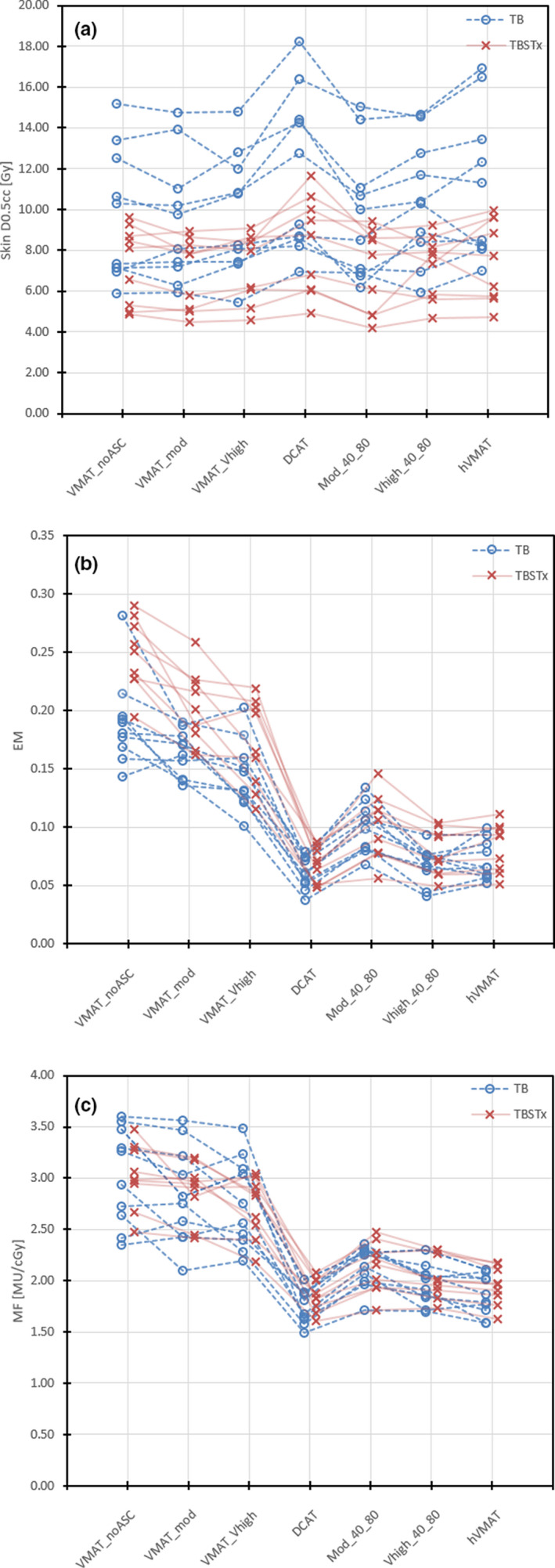
(a) Skin D0.5cc for each plan type and beam model. The skin dose for the TB model is systematically higher for all plan types. (b) Edge metric (EM) for each plan type and beam model. The EM for the TBSTx model is systematically higher than for the TB model, but this difference decreases as the EM decreases. (c) Modulation factor for each plan type and beam model. There is negligible difference in modulation factors between the two models in each plan type.

Overall, there were no data to suggest a significant dosimetric difference between the TB and TBSTx model; however, there were some noteworthy exceptions. The skin D0.5cc using the TBSTx model was systematically lower than the TB model for all plan types, as depicted in Fig. 1(a). This is likely attributed to the TB patients having more peripheral lesions, closer to the Skin contour, since the data did not suggest that the TB plans were more complex/modulated overall. Rather, the TBSTx EM was systematically higher than the TB model [Fig. 1(b)], in particular for the more modulated plan types. This was expected since the TBSTx plans use a greater number of leaf pairs to define each aperture, and thus introduce more leaf edges to the EM calculation. Despite this, the mean EMs for the DCAT, MU objective, and hVMAT plans are comparable for both models (see Table 2).

**Table 2:** Comparison of average EM for each beam model by plan type.

Plan type	TB EM Avg (±1SD)	TBSTx EM Avg (±1SD)
VMAT_noASC	0.18 ± 0.02	0.25 ± 0.03
VMAT_mod	0.16 ± 0.02	0.20 ± 0.03
VMAT_Vhigh	0.14 ± 0.02	0.17 ± 0.04
DCAT	0.06 ± 0.01	0.07 ± 0.02
Mod_40_80	0.10 ± 0.02	0.10 ± 0.03
Vhigh_40_80	0.07 ± 0.01	0.08 ± 0.02
hVMAT	0.07 ± 0.01	0.08 ± 0.02

Due to the open apertures, DCAT is the most robust to tumor motion of the plans investigated in this study, but the data in Table 1 shows that DCAT is generally dosimetrically inferior to VMAT plan types. The robustness metrics for the MU objective and hVMAT plans, however, were comparable to that of DCAT, as depicted in Figs. 1(b) and 1(c). The statistical testing confirmed that both the hVMAT plan and MU objective plan with “Very High” ASC showed no significant difference to DCAT (with the MU objective plus “Moderate” ASC only slightly different) in the robustness metrics.

## 4 Discussion

**Fig. 2:**
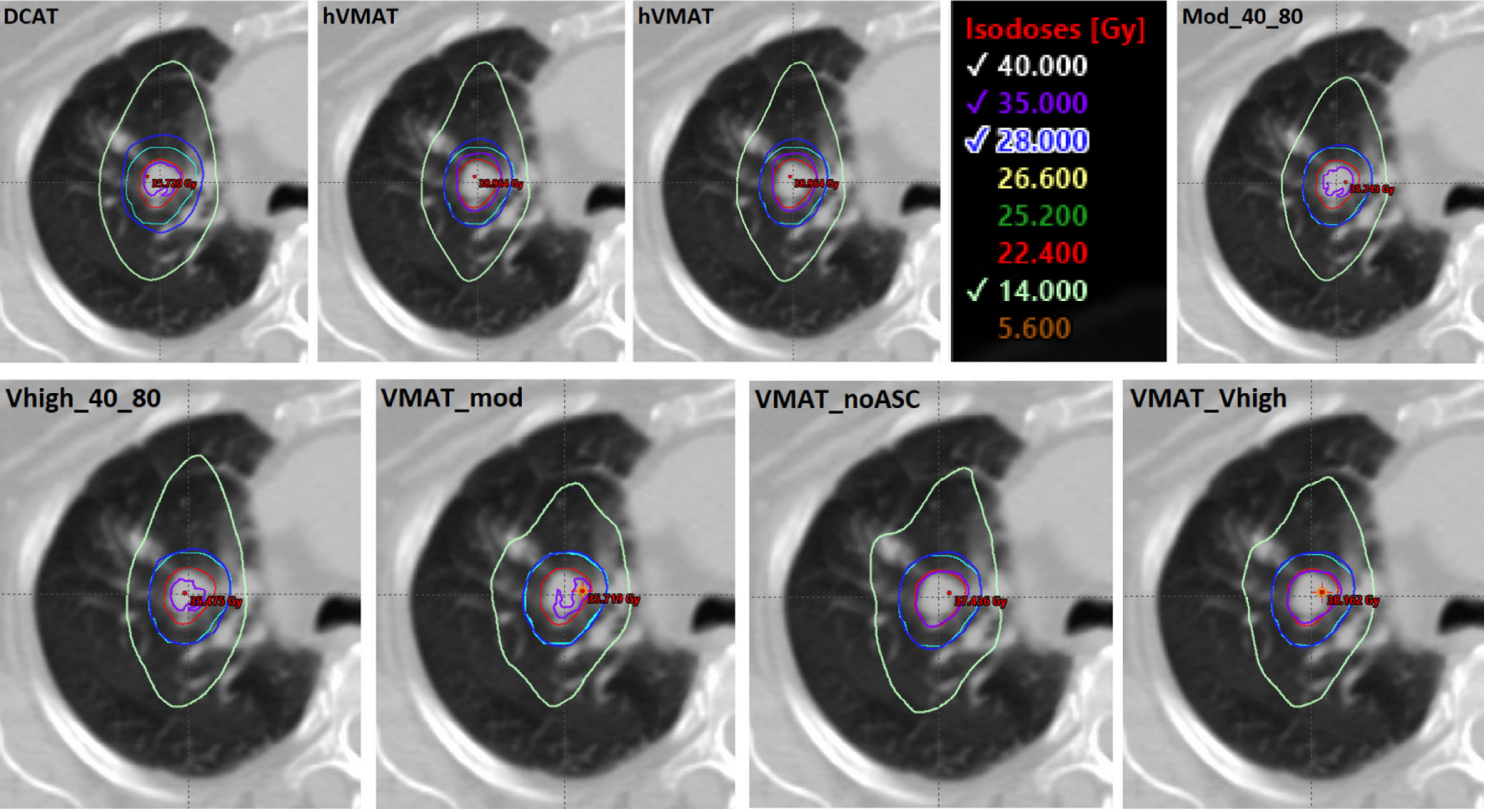
Example isodose distributions at the isocenter (internal target volume in red, planning target volume in cyan) for each plan type for the same patient.

The VMAT with ASC set to “None” plan type was chosen as the benchmark to illustrate the maximum achievable dosimetric plan quality, allowing the optimizer the most modulation. The results suggest that all plans tested have significantly less modulation than these unconstrained VMAT plans, and there is little dosimetric trade‐off in terms of OAR doses. When it comes to target dose and conformity, however, there is some compromise for penalizing modulation (see Fig. 2). All DCAT, MU objective and hVMAT plans showed deviations from the benchmark plan in the ITV D2%, CI100, and CI50 quality metrics; however, the absolute outcomes of these metrics are clinically acceptable in all VMAT plans (Table 1).Depending on the patient specific tumor to OAR geometry, this dosimetric trade off may not be appropriate and other options to reduce susceptibility to interplay effects may need to be taken.

While the EM is strictly speaking a measure of aperture irregularity, it can be used as a measure of VMAT aperture openness by comparison to the DCAT result (Table 1). In order for two plans to have the similar EM they must have a similar ratio of open to partially blocked control points when MU weight is considered. Since DCAT plans have only fully open apertures, a VMAT plan with a comparable EM must have negligible partially blocked, irregular apertures. This conclusion is consistent with the results of measurement‐based interplay studies in the literature.^9^, ^11^ In the context of the small fields used in SABR, if the total MU (or MF) for both plans are also similar, then they must have similar openness. In this way, we can be confident that the EM and MF are useful as a proxy for aperture openness. The use of the MU objective as described (maximum MU = 40% of original, strength = 80) in combination with the “Very High” ASC may therefore be appropriate for single fraction lung SABR treatments using FFF beams at high repetition rate, as the apertures may be sufficiently open to increase robustness to the interplay effect.

## 5 Conclusion

We have demonstrated significant reductions in modulation complexity through use of aperture shape controller and monitor unit restriction for lung SABR VMAT plans. These reductions were achieved with minimal dosimetric penalty, and may increase robustness to respiratory motion interplay effects in single fraction SABR with high dose rates observed with FFF beams.

## Conflict of interest

Dr. Nicholas Hardcastle receives research funding for an unrelated project from Varian Medical Systems.

## Appendix A

See Table A1.

**Table A1:** Combination of metrics trailed on first 10 TBSTx patients.

Plan type	ASC	MU objective	
		Max MU	Strength
DCAT	NA	NA	NA
VMAT	None, moderate, high, very high	NA	NA
hVMAT	Moderate, high, very high	NA	NA
VMAT with	Moderate, high, very high	40%	80
MU objective	Moderate, high, very high	60%	95
	Moderate, high, very high	70%	100
